# Tolerance and durability of abacavir/lamivudine (ABC/3TC)-containing regimens: results from a large prospective French cohort

**DOI:** 10.1186/1758-2652-13-S4-P44

**Published:** 2010-11-08

**Authors:** L Cuzin, C Allavena, L Levy-Bachelot, MA Valantin, H Mellliez, P Pugliese, I Poizot-Martin, C Duvivier, L Finkielsztejn, S Abel

**Affiliations:** 1 CHU Toulouse, COREVIH Midi Pyrénées Limousin, Toulouse, France; 2 University Hospital Nantes, Infectious Diseases Department, Nantes, France; 3 Laboratoire GlaxoSmithKline, Marly-le-Roi, France; 4 AP-HP Pitié-Salpêtrière Hospital, Paris, France; 5 Centre hospitalier de Tourcoing, Service Universitaire des Maladies infectieuses, Tourcoing, France; 6 Nice University Hospital, Infectious Disease Dpt, Nice, France; 7 Hôpital Sainte-Marguerite, Service d'Immuno-Hématologie Clinique, C.I.C., Marseille, France; 8 Institut pasteur, Centre Médical de l’Institut Pasteur, Paris, France; 9 ViiV Healthcare France, Département Médical, Marly le Roi, France; 10 CHU de Fort-de-France, Infectious Disease Dpt, CIC-EC 802 Antilles Guyane, Fort-de-France, Martinique (French)

## Methods

Patients (pts) were selected from the Dat'AIDS French prospective cohort if they were prescribed an ABC/3TC containing regimen for the first time between 01/01/2004 and 31/12/2007 and were still actively followed on 31/05/2008 as to ensure sufficient follow-up. All causes of treatment discontinuation were recorded, as well as immuno-virological data and cardiovascular events (CE) during follow-up.

## Results

Among the 1704 pts included in the study (male 70%, mean age 43 years, HBV or HCV co-infection 24.1%) 407 (24%) were antiretroviral (ARV) naïve, 696 (41%) were virologically controlled on ARV treatment (switch), and 601 (35%) were on treatment with detectable VL (failure) at time of ABC/3TC initiation. Previous treatment with 3TC was noted in 92% of the pretreated pts. With a median duration of follow-up of 496 days, the population represents 2636 pts-year.

Overall 565pts (33%) discontinued ABC/3TC combination during follow-up (36%, 24%, and 42% of the naive, switch and failure groups, respectively). Reasons for discontinuation were poor tolerance in 41% of the cases, including suspected hypersensitivity (HSR) in 4% of the overall population, treatment failure in 20%, and other causes in 39%. This distribution was not different if pts received either a NNRTI (20% of the pts) or a boosted protease inhibitor (46%) in their regimen. Median time to discontinuation was 4.3 years overall and less than one month in case of suspected HSR. Discontinuation for bad tolerance was observed in 38%, 54%, and 35% of the naïve, switch and failure populations respectively, whereas treatment failure was responsible for discontinuation in 15%, 10%, and 29% of the same populations. Major CE were reported in 21 cases (0.08% py). Death was recorded in 27 cases, 9 deaths being AIDS related, 6 related to liver diseases, 6 to cancer, 2 to vascular accidents (1 cardiac, 1 neurological), 1 accidental, and 3 unknown. At M24, probability of still receiving ABC/3TC was 62%, 77%, and 60% respectively for the defined groups, and VL on treatment was below detection for 86%, 90%, and 71% of them, respectively.

## Conclusion

In this population of pts who received ABC/3TC containing regimens before HLA screening was routinely available, treatment was maintained with virological success for more than 2 years. Poor tolerance was the main reason for early discontinuation, and was not different if the pts received either NNRTI or boosted PI in their regimen. CE were rare.

